# Matrix Recruitment and Calcium Sequestration for Spatial Specific Otoconia Development

**DOI:** 10.1371/journal.pone.0020498

**Published:** 2011-05-31

**Authors:** Hua Yang, Xing Zhao, Yinfang Xu, Lili Wang, Quanyuan He, Yunxia Wang Lundberg

**Affiliations:** 1 Vestibular Neurogenetics Laboratory, Boys Town National Research Hospital, Omaha, Nebraska, United States of America; 2 Verna and Marrs McLean Department of Biochemistry and Molecular Biology, Baylor College of Medicine, Houston, Texas, United States of America; University of California, Merced, United States of America

## Abstract

Otoconia are bio-crystals anchored to the macular sensory epithelium of the utricle and saccule in the inner ear for motion sensing and bodily balance. Otoconia dislocation, degeneration and ectopic calcification can have detrimental effects on balance and vertigo/dizziness, yet the mechanism underlying otoconia formation is not fully understood. In this study, we show that selected matrix components are recruited to form the crystal matrix and sequester Ca^2+^ for spatial specific formation of otoconia. Specifically, otoconin-90 (Oc90) binds otolin through both domains (TH and C1q) of otolin, but full-length otolin shows the strongest interaction. These proteins have much higher expression levels in the utricle and saccule than other inner ear epithelial tissues in mice. *In vivo*, the presence of Oc90 in wildtype (wt) mice leads to an enrichment of Ca^2+^ in the luminal matrices of the utricle and saccule, whereas absence of Oc90 in the null mice leads to drastically reduced matrix-Ca^2+^. *In vitro*, either Oc90 or otolin can increase the propensity of extracellular matrix to calcify in cell culture, and co-expression has a synergistic effect on calcification. Molecular modeling and sequence analysis predict structural features that may underlie the interaction and Ca^2+^-sequestering ability of these proteins. Together, the data provide a mechanism for the otoconial matrix assembly and the role of this matrix in accumulating micro-environmental Ca^2+^ for efficient CaCO_3_ crystallization, thus uncover a critical process governing spatial specific otoconia formation.

## Introduction

Otoconia, like bone, are a result of ordered deposition of inorganic calcium carbonate (calcium phosphate in bone) crystallites onto a pre-formed framework of organic matrix of glycoproteins. The organic matrix of otoconia is composed of a predominant glycoprotein plus low-abundant glycoproteins (collectively called otoconins) and proteoglycans [1]–[10]. While bone consists of over 200 proteins, otoconia in mice only have about 8 components identified to date. The predominant mammalian otoconial protein is otoconin-90 (Oc90) [7]; [8], and the minor murine otoconins are otolin-1 (aka otolin) [10], osteopontin [11]–[13], fetuin-A (aka countertrypin) [6]; [10], Sparc-like 1 (secreted protein acidic and rich in cysteine, aka Sc1 or hevin) [6]; [9], and possibly Sparc, DMP1 (dentin matrix protein) and α-tectorin [9]. The proteoglycan in murine otoconia is keratin sulfate proteoglycan (KSPG)[9].

Notably, Oc90 is not related to the predominant bone matrix protein (collagen I in cortical bone and collagen II in cartilage) or any other known matrix protein, but rather, it has two domains resembling secretory phospholipase A2 (sPLA2) [7]; [8] with 31 and 35% amino acid identity (45% and 48%, respectively, with conservative variations counted). The predicted tertiary structure of either domain of Oc90 is almost super-imposable to that of sPLA2 [9]. However, Oc90 does not have the catalytic activity of sPLA2 due to substitutions of a few of the critical residues [5]; [8]. Experimental structure-function analysis has not been done to demonstrate the functional significance of conservation of the sPLA2 structure, but the critical features have been analyzed and modeled [9] and is re-capitulated in the Discussion section of this report.

Interestingly, the low abundance otoconins either play critical roles in bone calcification, or belong to the family of essential bone proteins. For example, otolin contains an N-terminal collagen-like domain (also called triple helix or TH domain) and a C-terminal globular (gC1q) domain (also called non-collagenous or NC1 domain) [14], and belongs to the collagen X family and the C1q super-family. Collagen X is a cartilage-specific collagen important for the onset of cartilage mineral deposition and the normal distribution of cartilage extracellular matrix components, especially proteoglycans, within the growth plate of long bones, ribs, and vertebrae [15]–[20]. The TH domain in collagen X contains Gly-X-Y repeats that are mandatory for the formation of a collagen triple helix; even a single Gly substitution can destabilize the triple helix through a local disruption in hydrogen bonding [21]. C1q members are the target recognition proteins of the classical complement pathway. C1q proteins bind through its collagen-like domain to surface receptors of fibroblasts and to adhesive elements of extracellular matrix including fibronectin, proteoglycans and laminin [18]; [22]; [23]. gC1q is highly conserved, distinguishes the C1q sub-families and is well known for its flexibility and versatility in ligand recognition [24]; [25].

Although only partial details of otoconia formation have been revealed, this much is clear: the ultrastructure and function of the otoconial matrix in regulating crystal growth are similar to that in bone. That is, the matrix proteins in both systems form a fibrous network [10]; [26]; [27] to regulate the growth of the mineral crystals but neither is involved in the initial mineral accretion [10]; [28]. In bone, collagens interact with other important matrix proteins such as Sparc, osteopontin, bone sialoprotein, fibronectin, vitronectin [23]; [29]–[31]. In otoconia (or fish otolith), evidence suggests that the critical otoconins may also interact with each other to form the organic framework for efficient crystallization [Lundberg et al., Association for Research in Otolaryngology 2009 Abstract #935] [10]; [32]; [33]. In the absence of Oc90, the otoconia organic matrix, particularly otolin, is absent (or below detection level) and the efficiency of crystal formation is reduced by 50% [10]. Therefore, in the present study, we examined the interactions of Oc90 with the domains of otolin to gain insight on how the otoconial matrix is assembled. We also tested whether Oc90 binds KSPG as does sPLA2 to proteoglycans (particularly heparin sulfate proteoglycan, as HSPG) [34]; [35]. Given the known interaction between C1q proteins and proteoglycans, we tested whether the C1q-containing otolin interacts with KSPG as well.

While the overall design of matrix formation and subsequent crystallite deposition is similar between otoconia and bone, details of the calcification processes differ significantly between the two systems. For example, a few minor otoconins (i.e. osteopontin and fetuin-A) that are dispensable for otoconia formation play critical roles in bone matrix calcification [10]. In bone, osteopontin is a major non-collagenous protein and influences the organic matrix over mineral content and limits bone crystal sizes [21]; [28]. Its role in bone is similar to that of Oc90 in otoconia [10].

More importantly, unlike the bone milieu, the endolymph surrounding otoconia has an extremely low concentration of Ca^2+^, making mechanisms of otoconia formation perplexing. In order for CaCO_3_ to crystallize, the otoconia organic components must be able to sequester Ca^2+^ to efficiently raise its micro-environmental concentration, which was tested in the present study. In addition, the expression of some critical protein(s) must be restricted to or higher in the utricle and saccule than other inner ear tissues to account for the spatial specificity of otoconia development. Here we used both *in vivo* and *in vitro* approaches to examine how some critical otoconins participate in otoconia formation and whether they have higher expression levels in the otolithic organ.

## Materials and Methods

### Mice

Oc90 targeted mice were previously generated [10] and backcrossed to C57Bl/6J (B6) mice. Unless otherwise indicated, the reported results were all obtained from homozygous mutants. All animal procedures were approved by the Institutional Animal Care and Use Committee at the Boys Town National Research Hospital in accordance with federal and international guidelines (approval numbers 07-01 and 10-02).

### Construction of expression vectors

Transcripts for full-length and domains of proteins were amplified by RT-PCR of postnatal mouse inner ear tissues, and unidirectionally cloned into pAAV-IRES-hrGFP (pOc90-FLAG) and pcDNA3.1 (pOtolin, pOtolin-TH, pOtolin-C1q). GFP is co-transcribed but not fused with Oc90. For stable transfection, the full-length Oc90 transcript (stop codon omitted) was also unidirectionally cloned into pTracer-EF/V5-His(C) (pOc90-His) using the same restriction sites as below. pOtolin-TH contained residues 1–342, and pOtolin-C1q contained residues 1–60 fused with residues 343–482. An existing working antibody recognizes the N-terminal 17 amino acids of mature otolin (residues 24–40).

The following primers were used to amplify the respective transcripts: *Oc90* forward, 5′-g*gaattc*caccATGATTATGCTGCTCATGGTCGGT-3′; Oc90 *reverse*, *5′- gctcgagTTTCCCACCGAGGGGTCTGGCCC-3′; Otolin forward, 5′-gggatccaccATGTGGATA TTTTCTTCGCTTTGTGCTG-3′; Otolin reverse, 5′-ggaattcTGGTGACTTACTAAAAGTTTCCTCTGGGTACA-3′; Otolin-TH forward, 5′- cttggatccATGTGGATATTTTCTTCGCTTTG-3′; Otolin-TH reverse, 5′- ttagaattcttaGGCCTCGCCTTTGGAACCCTT-3′; Otolin signal peptide forward, 5′-cttggatccATGTGGATATTTTCTTCGCTTTG-3′; Otolin signal peptide reverse, 5′-atcgaattcCGTATGGACGGTTTCTTCT-3′; Otolin-C1q forward, 5′-agagaattcACACAAGTCCCACAGTCGGC-3′; Otolin-C1q reverse, 5′-cgtgatatcTTATGGTGACTTACTAAAAG-3′. Nucleotides in lower cases were non-endogenous sequences to create restriction sites (italicized) for cloning, and restriction enzymes used were EcoR I and Xho I for Oc90; BamH I and EcoR I for Otolin; BamH I and EcoR I for Otolin-TH; BamH I, EcoR I and EcoR V for Otolin-C1q. Constructs were confirmed by DNA sequencing and Western blotting.*

### Anti-FLAG co-immunoprecipitation (co-IP)

HEK293 cells (ATCC, Manassas, VA, USA) were cultured at 37°C in DMEM containing 4.5 g/L glucose, 100 U/ml penicillin, 4 mM L-glutamine and 10% fetal bovine serum. The cells were transiently co-transfected with pAAV-Oc90-FLAG-GFP + pOtolin (or pOtolin-His or Otolin domains) using FuGENE® HD transfection reagent (Roche, Indianapolis, IN) and maintained for 48 hrs before harvest for immunoprecipitation.

Co-IP was performed based on a protocol for anti-FLAG M2 affinity gel by Sigma (St. Louis, MO). Briefly, cells were lysed on ice with gentle hypotonic buffer (10 mM Tris-HCl pH 7.5, 10 mM NaCl, 2 mM EDTA, 0.5% Triton-X-100 and 0.5% protease inhibitor cocktail) with gentle rocking for 20 min. After addition of 150 mM NaCl (final concentration), samples were centrifuged at 13,000 rpm (16,000 g) for 10 min at 4°C, and supernatants were collected. Aliquots (400 µl) of supernatants were incubated under constant agitation with 30 µl anti-FLAG-M2 bead slurry (Sigma) for 6 h at 4°C. Beads were washed 3 times with TBS, and proteins were stripped using 60 µl elution buffer (0.1 M glycine HCl, pH 3.5) at room temperature and neutralized with 6 µl buffer (0.5 M Tris-HCl pH 7.4, 1.5 M NaCl). After centrifugation at 5,000 g for 30 sec, proteins were analyzed by Western blotting as described below. The primary antibodies were used at 1∶200 for otolin and 1∶1000 for Oc90. The secondary antibody, a horseradish peroxidase (HRP)-conjugated goat anti-rabbit IgG, was used at 1∶10,000 dilution (Sigma).

The interaction of Oc90 and otolin with KSPG was examined as described in the supplemental Method S1.

### Western blotting

Proteins from utricular and saccular epithelia, otoconia and cultured cells were extracted at 4°C overnight in 10 µl of lysis buffer containing 40 mM Tris, 4% CHAPS, 8 M urea, 10 mg/ml DTT and 0.15 M EDTA. After removal of EDTA and salts using a Centricon column (Millipore, Billerica, MA, USA), protein concentrations were determined using a Micro-BCA (bicinchoninic acid) Protein Assay Kit (Pierce, Rockford, IL, USA) following the manufacturer's protocol. BSA standards were included to calculate sample protein concentrations.

An aliquot of each sample containing an equal amount of total protein was mixed with 2X sample loading buffer (0.13 M Tris-HCl, 20% glycerol, 46 mg/ml SDS, 0.2 mg/ml bromophenol blue, 20 mg/ml DTT), boiled for 5 minutes, loaded onto a 4–15% gradient Tris-SDS gel (Bio-Rad, Hercules, CA, USA), electrophoresed at 100 V in running buffer (25 mM Tris pH 8.3, 192 mM glycine, 0.1% SDS) for 1–2 hours, and transferred to a PVDF membrane (Millipore) at 100V for 1 hour. The blot was washed once with TBST buffer (50 mM Tris, 150 mM NaCl, 0.1% Tween-20) and treated with 1% blocking buffer (Roche Applied Science, Indianapolis, IN, USA) overnight at room temperature. The membrane was incubated with primary antibodies at appropriate dilutions for 3 hours followed by 3 washes in TBST buffer, and incubated with a specific peroxidase-conjugated secondary antibody (Sigma) at 1∶10,000 dilution for another hour. After 3 washes, an ECL detection solution (Pierce, Rockford, IL) was added for 1 minute, and the blot was exposed to a Kodak X-ray film and imaged using the Canoscan 8400F imaging station.

### Fluorescent immunostaining

Frozen tissue sections (9 µm) were treated with blocking solution containing 5% goat serum and 0.2% Triton-X-100 at room temperature for 30 minutes. Primary antibodies were added at a dilution of 1∶100 to 1∶600, and incubated at 4°C overnight. After 3 washes in 1xPBS, Alexa-488 or 568 (Molecular Probes, Carlsbad, CA, USA) conjugated secondary antibodies were added at a dilution of 1∶600, together with DAPI (1∶10000), and incubated at room temperature for 2 hours in the dark. Either null tissues or non-immune sera (instead of primary antibodies) were used as negative controls in the preliminary experiment. Slides were mounted in Fluoromount-G and signals were viewed using a Zeiss LSM 510 confocal microscope.

### Real-time quantitative RT-PCR (qRT-PCR)

Primers and probes for qRT-PCR were purchased from ABI (Applied Biosystems, Foster City, CA, USA), and qRT-PCR was done according to the manufacturer's protocol with minor modifications based on cDNA samples and primers/probe conditions. The ABI catalog number for Oc90 primers/probe was Mm01200961_m1 and otolin Mm01222538_m1.

Total RNA was prepared using Trizol (Invitrogen), and an equal amount of RNA from each tissue (50 to 100 ng) was reverse transcribed using the SuperScript III first-strand synthesis system (Invitrogen). 40 cycles of amplification was achieved in a 96-well plate consisting of TaqMan Gene Expression Assay, TaqMan Universal PCR Master Mix and cDNA. The standard mode of an ABI Prism 7900HT Sequence Detection System was used, and β-actin was amplified in parallel as an endogenous control to standardize the amount of sample added to a reaction and for the quantification of the relative gene expression within and between samples. All reactions were performed in triplicates.

### Cell culture and stable transfection

NIH/3T3 cells (ATCC) were cultured in DMEM containing 4.5 g/L glucose, 100 U/ml penicillin, 4 mM L-glutamine and 10% fetal bovine serum. Conventional culture condition was used at 37°C in a humidified incubator supplemented with 5% CO_2._ The cells were transfected with pOc90-His and pOtolin, alone or together, and with the empty vector pcDNA3.1 or pTracer™-EF/V5-His using FuGENE® HD (Roche Applied Science, Indianapolis, IN). Stably transfected clones were selected over a two-week period using 400 µg/ml Zeocin for pOc90-His, or 800 µg/ml G418 for pOtolin. Selected clones were then expanded and analyzed for calcification potential as described below.

### Induction of calcification

To induce ECM (extracellular matrix) mineralization, stable NIH/3T3 clones expressing pOc90-His, pOtolin, pOc90-His + pOtolin, pTracer™-EF/V5-His and pcDNA3.1 were treated with 0.5 mM Ca^2+^ (CaCl_2_) and 2 mM Pi (Na_2_HPO_4_) in growth media to provide a source of inorganic components for mineralization. Phosphate, instead of carbonate, was used so as not to alter the optimal pH and CO_2_ content. After 5 days of induction, cells were fixed with 4% PFA, and stained with Alizarin Red S (ARS) (Chemicon, Billerica, MA). The effects of various Ca^2+^ and Pi concentrations were tested in the preliminary experiments to determine the optimal condition at which the effects of Oc90 and otolin were best differentiated from empty vectors. In order to minimize variations caused by the effect of cell density on calcification, cells were seeded at a density (2×10^4^ cells in each well of the 24-well plate) so that each well was grown to confluence when calcification was examined on the 6^th^ day. The supplemental Ca^2+^ and Pi reduced the proliferation rate of all cells, whether they were transfected or not. Calcification nodules and total cells were photographed and counted for each 20x view field under an inverted microscope (approximately 1.2 mm^2^); averaged ratios of nodules/total cells were compared among transfectants and the vector using the Student's t-test.

### Ratiometric calcium measurement

The ECM-Ca^2+^ content of Oc90 null and wt tissues was determined using the ratiometric dye, Fura-2 (Molecular Probes, Eugene, OR). Fura-2 was used for the particular goal because micro-electrodes only measure free Ca^2+^, and Alizarin Red staining for calcium deposits (calcium phosphate/carbonate) is not sensitive enough to stain the early stage of crystal seeds [10], nor does it detect immobilized Ca^2+^.

Both fixed and unfixed tissues were tested for comparison. Epithelia from the utricle+saccule, canal+ampulla, and cochlea were dissected in artificial endolymph with or without fixation, were then soaked at 4°C overnight in 5 μl cell fura-2 pentapotassium salt (Invitrogen) in Ca^2+^ free Ringer solution. The cell- and matrix-free supernatants were then diluted 10 fold with a Ca^2+^ free Ringer solution, and an aliquot of test solution was placed in a sealed glass pipette and imaged using a cooled CCD camera (SensiCam, Cooke Corp., La Jolla, CA) on an upright fixed stage microscope (Nikon E600FN, Garden City, NY). Axon Imaging Workbench (AIW 2.2, Axon Instruments) was used to control the camera, filter wheel (Lambda 10-2, Sutter Instruments, Novato, CA), and image acquisition. [Ca^2+^] was calculated from the ratio of light emitted at 510 nm during alternate excitation by 340 and 380 nm light [36]. Calibration constants (standard curve) were determined using a series of highly buffered Ca^2+^ test solutions (Molecular Probes), and Ca^2+^ concentrations eluted from the ECM of each tissue from each animal were determined using the standard curve. Occasional statistical outliers (> +2SD or < −2SD) were excluded from further analysis.

Due to the high sensitivity of the assay, there were some variations in the absolute readings between experiments done on different days, therefore, measurements from the same batch (done on the same day) consisted of both Oc90 wt and null genotypes of each age group to obtain wt/null ratios. Wt/null ratios were used also because the endolymph volume covering the mouse utricular and saccular macula ECM is unknown and the Ca^2+^ is presumably immobilized in the ECM, making it impossible to calculate the absolute concentrations of micro-environmental Ca^2+^.

### Data analysis

Statistical analysis was performed using the Student's t-test. P values of less than 0.05 were considered significant.

### Molecular modeling

To model the C1q domain of otolin (Swiss-Prot id Q4ZJM7), we used I-TASSER server [37] to search mouse otolin-C1q domain sequence (amino acids 343–478) against PDB database [http://www.rcsb.org/pdb/home/home.do]. Ten templates from three structures (PDB id 1a05A, 1cnzB and 1cnzA) were used to build a hybrid homology model. We then used GROMACS [38] to optimize the structure by molecular dynamics (MD) simulations via three steps: (1) energy minimization in 1.2 ps; (2) position-restrained molecular dynamics in 50 ps to soak the water/ion molecules into the domain structure; and (3) molecular dynamics simulation in 1000 ps. All simulations were done in the condition of 150 mM NaCl solution at 37°C. Modeling of the TH domain of otolin was similarly done.

## Results

### Oc90 interacts with both domains of otolin but prefers the full-length protein

**Figure 1:**
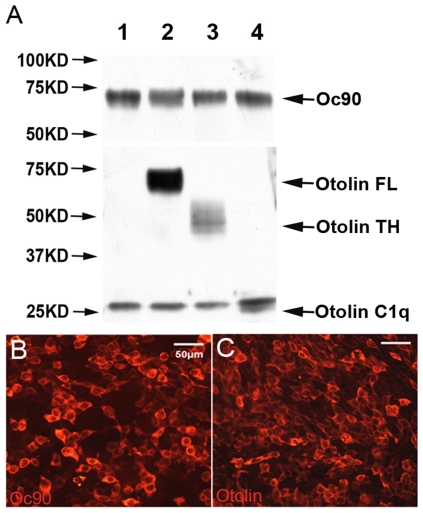
Co-immunoprecipitation (co-IP) of Oc90 with otolin or its domains. HEK293 cells were transfected with full-length Oc90-FLAG, otolin and domains of otolin. Shown in (**B, C**) are Oc90-FLAG and otolin detected by immunostaining. In (**A**), protein products were co-immunoprecipitated using anti-FLAG agarose beads. The lanes are co-IP with: 1) empty vectors, 2) full-length (FL) otolin, 3) otolin-TH domain, and 4) otolin-C1q domain.

To test whether Oc90 interacts with otolin to form the organic matrix of otoconia and to identify the interacting domains, we performed co-immunoprecipitation (IP). Transfection of HEK293 cells with expression constructs containing full-length Oc90 (Oc90-FLAG), full-length and individual domains of otolin (TH and C1q) yielded high expression efficiency (Figure 1B, 1C). Protein products were co-precipitated using anti-FLAG agarose beads and washed under stringent conditions as described in Materials and Methods. Constituents eluted from the beads were analyzed by Western blotting after separation by 4–15% SDS-PAGE (Figure 1A). Antibodies against the N-terminal regions of Oc90 and otolin [10] were used for detection. Oc90 interacts strongly with full-length otolin (lane 2 in Figure 1A), and both domains of otolin participate synergistically in this interaction (lanes 3 and 4 in Figure 1A). The requirement of both otolin domains is consistent with the finding that the C1q domain of other related proteins is responsible for partner recognition and the TH domain for strengthening the interaction [22]. Both the TH and C1q domains have a high affinity for proteoglycans and Ca^2+^ as well [39]. As is the case with the sPLA2-like domains in Oc90 [8]; [9], the functional domains of otolin (TH and C1q) are extremely conserved throughout evolution [32].

**Figure 2:**
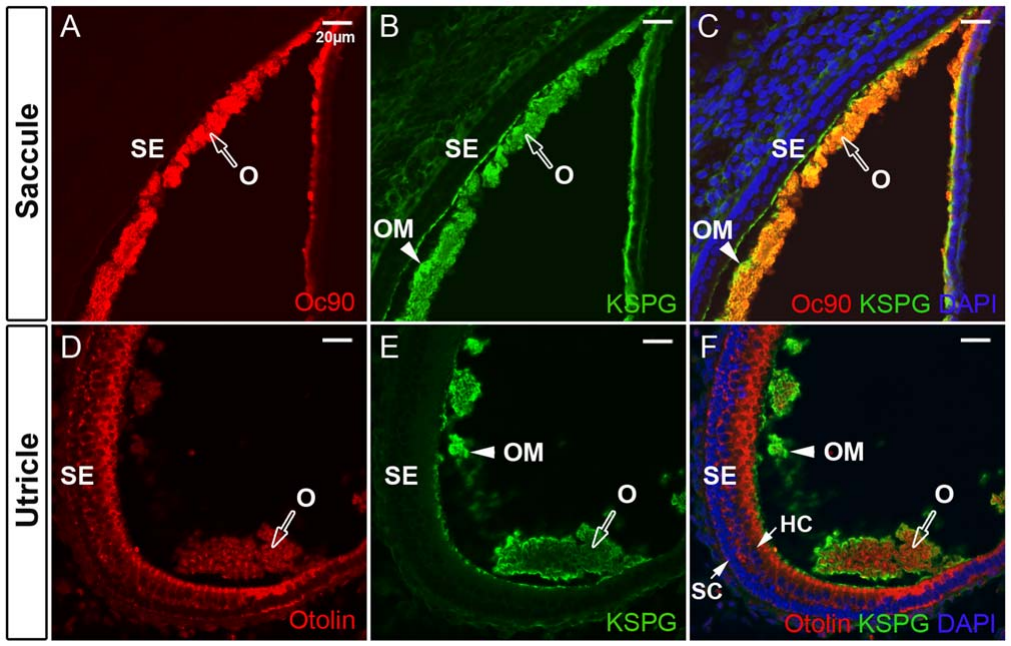
Relative presence of Oc90, otolin and KSPG in neonatal B6 otoconia. (**A, C**) Abundant presence of Oc90 in otoconia crystals (labeled as ‘O’). (**D, F**) Otolin antibody showed staining in both the sensory epithelium (labeled as ‘SE’) and otoconia. (**B, C, E, F**) KSPG had a low level in the crystals but showed intense staining in the otoconia membrane (labeled as ‘OM’). O, otoconia; OM, otoconia membrane; SE, sensory epithelium; HC, hair cells; SC, supporting cells.

As the main Xenopus saccular otoconin Oc22 [40] only has one sPLA2-like domain and does not have the N- or C-terminal region of Oc90, we predicted that a single sPLA2-like domain of Oc90 will be able to bind otolin and, therefore, did not test each Oc90 domain alone. Instead, we further tested whether Oc90 or otolin interacts with KSPG, another otoconia component of low to intermediate abundance. Because Western blotting of KSPG tends to produce a smear due to non-homogeneous degree of glycosylation, we used protein A agarose beads to bind an antibody for KSPG, followed by incubation with KSPG and Oc90 (or otolin) extracted from otoconia, vestibular epithelia or transfected cells. As shown in the supplemental Figure S1, the co-IP reaction produced a weak band of Oc90 (or otolin) in the reaction containing anti-KSPG+KSPG+Oc90 (or otolin), but not in the reaction containing mouse IgG+KSPG+Oc90 (or otolin) control, therefore, the reaction was likely specific albeit weak. KSPG is present in Oc90 null otoconia and vestibule in a way similar to that in wt tissues [9], further suggesting weak interactions between these two components. The weak interaction between Oc90 (or otolin) and KSPG is also consistent with the observation that KSPG is low in the crystals but abundant in the otoconial membrane (OM) (Figure 2), whereas the expression of both Oc90 and otolin is higher in the crystals than the OM. Based on our previous mass spectrometry data [10], Oc90 is much more abundant in the crystals than otolin, which is evident here by the fluorescence intensity in the Figure 2. Different tissue sections were used for Oc90 and otolin immunostaining simply because of their availability at the time of the experiment; based on previous observation [10], the utricle and saccule have similar patterns of presence of the two proteins.

### Oc90 and otolin have much higher expression levels in the utricle and saccule than the semi-circular canals or cochlea

**Figure 3:**
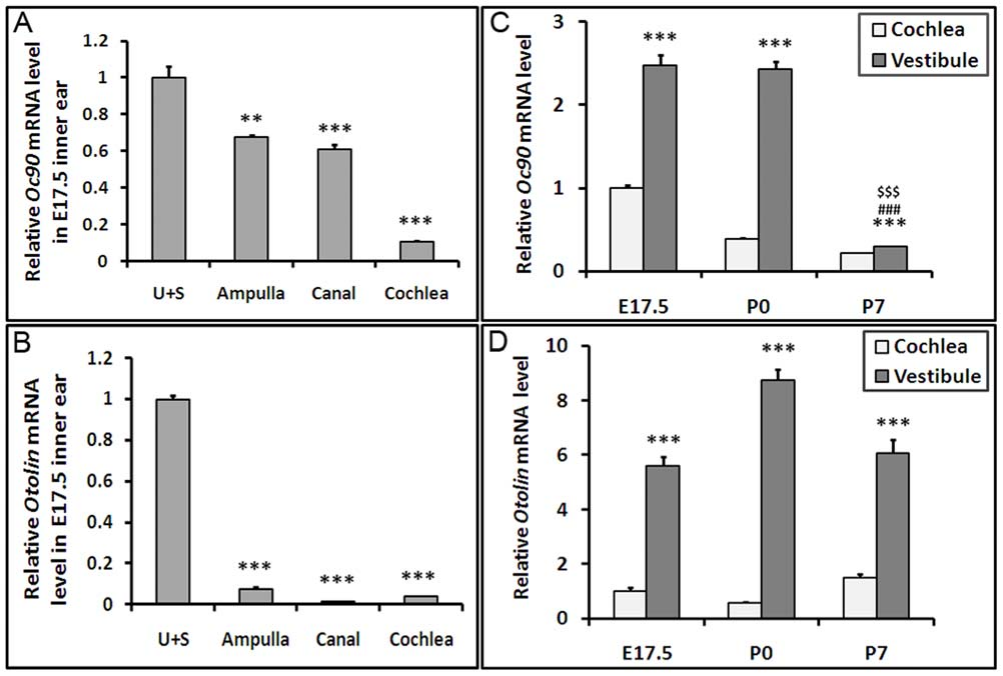
Oc90 (A, C) and otolin (B, D) expression levels in murine inner ear. Quantitative real-time RT-PCR (qRT-PCR) showed that both Oc90 and otolin had significantly higher expression levels in the utricle and saccule than the ampulla, canal or cochlea in wt mice. The mRNA levels were normalized to that of β-actin (relative mRNA level), and then different tissues were compared to the utricle and saccule whose relative mRNA level was set to 1 (**A, B**). In (**C, D**), the relative mRNA level in the E17.5 cochlea was set to 1. The vestibular samples in C and D mainly consisted of the utricle+saccule with a small part of the ampulla. ** and *** denote p<0.01 and 0.001, respectively, when the utricle+saccule are compared with other tissues (n = 3 for each type of tissue in each age group). ### and $$$ denote p<0.001 when P7 is compared with E17.5 and P0, respectively.

In order to examine whether Oc90 and otolin have higher expression levels in the utricle and saccule that may contribute to the spatial restriction of otoconia formation in these tissues, we performed qRT-PCR analysis of inner ear epithelial tissues dissected from E17.5 (embryonic day 17.5), P0 (postnatal day 0) and P7 wt mice. As shown in Figure 3, the expression levels of Oc90 and otolin were 10 to 20 times higher in the utricle and saccule than the cochlea at E17.5 (p<1.4 X10^−6^ and p<1.6X10^−6^, respectively, n = 3, indicated by *** in Figure 3). The Oc90 mRNA level in the utricle and saccule drastically reduced at around P7 as compared with that at E17.5 and neonatal (P0) stages (p<7.4X10^−6^ and p<2.0X10^−6^, respectively, n = 3, indicated by ^###^ and ^$$$^ in Figure 3C). The vestibular samples in Figure 3C and 3D mainly consisted of the utricle+saccule with a small part of the ampulla. The changes in Oc90 mRNA levels at these stages coincide with the growth and the arrest of growth thereafter of otoconia. In comparison, the otolin mRNA levels show smaller changes across ages. This, together with the relatively late onset of otolin expression in the utricle and saccule, likely reflects a possible role of otolin in crystal growth and maintenance. In contrast, the early onset of Oc90 expression [7]; [8] reflects its role in crystal seeding and growth [10]. The expression of either gene in adult tissues was extremely low (data not shown).

### Oc90 (or Oc90-otolin complex) sequesters Ca^2+^ *in vivo* for optimal otoconia formation

In order for otoconia to form in the low [Ca^2+^] endolymph, participating proteins must be able to bind and sequester Ca^2+^ for efficient CaCO_3_ crystallization. Indeed, all of the otoconial proteins known to date have Ca^2+^-binding motifs or structural features. If so, there may be a higher matrix-Ca^2+^ content in the utricle and saccule in the presence of otoconial proteins such as Oc90 and otolin as compared to that in their absence. Furthermore, the matrix-Ca^2+^ may be higher in the utricular and saccular matrix where otoconia are formed and where otoconial proteins such as Oc90 and otolin have much higher expression levels, and lower in the ampulla/canals and cochlea where no otoconia are present under normal physiological conditions.

The difficulty lies in finding or developing a sensitive assay suitable for the minute tissue and matrix to be tested. Due to the high affinity of Fura-2 and the relatively low affinity of matrix proteins for Ca^2+^ [e.g., the affinity of fura-2 for Ca^2+^ is ∼0.14 µM, whereas the affinity of Col10a1 for Ca^2+^ is around 32 µM] [41], we were able to use fura-2 to extract the immobilized Ca^2+^ from the luminal surface and measure the Ca^2+^ content in the cell- and matrix-free supernatant. The pentapotassium salt of fura-2 is cell impermeant; nevertheless, we tested both fixed and unfixed tissues to detect any possible bias that may have arisen from Ca^2+^ outfluxes from intra-cellular sources. Not surprisingly, fixed and unfixed tissues gave similar measurements, likely because only Ca^2+^ in the extracellular solution was being examined.

**Figure 4:**
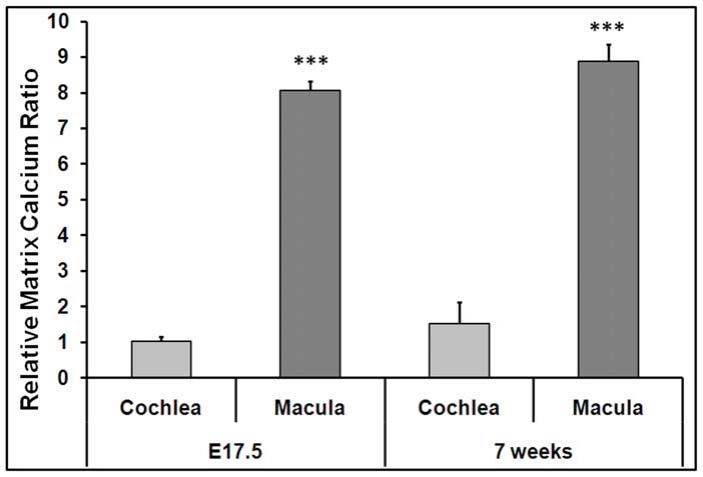
Oc90 sequesters Ca^2+^ *in vivo*. Oc90 wt/null ratios of matrix-Ca^2+^ content in E17.5 and 7 week-old mice as measured with a cell impermeant fura-2 calibration kit (n = 8–16 in each tissue, age and genotype group). *** indicates p<0.001 between measurements of the utricle+saccule vs. cochlea.

By applying the fura-2 calibration kit, we indeed detected the trend hypothesized above (Figure 4). In the figure, the Oc90 wt/null ratios were obtained by using the averaged [Ca^2+^] values (based on the *in vitro* solution volume of 50 µl) from each tissue, genotype and age group (n = 8–16 in each group). Oc90 wt/null ratios of matrix-Ca^2+^ content in E17.5- and 7-week-old mice were consistently 8–9 in the macular matrix, but there was no significant difference between the two genotypes in the cochlear matrix where both genotypes produced low measurements. A preliminary test of wt and null canals (including the ampulla ends) showed an outcome similar to that of the cochlea (data not shown). Maintenance of the macular matrix-Ca^2+^ content at the onset of murine adulthood, a stage considerably beyond the postnatal otoconia growth period (growth arrests at around P7), suggests a requirement for maintenance or possible potential for regeneration/neogenesis. Regardless of the gene expression levels after early postnatal stages, the matrix proteins are still present or even abundant (i.e. Oc90) [10] likely due to their stability.

From these data, we conclude that Oc90 (or Oc90-otolin complex) is important to sequester Ca^2+^ in the utricular and saccular ECM for spatial specific otoconia development in wt mice.

### Oc90 and otolin synergistically augment ECM calcification in cell culture

**Figure 5:**
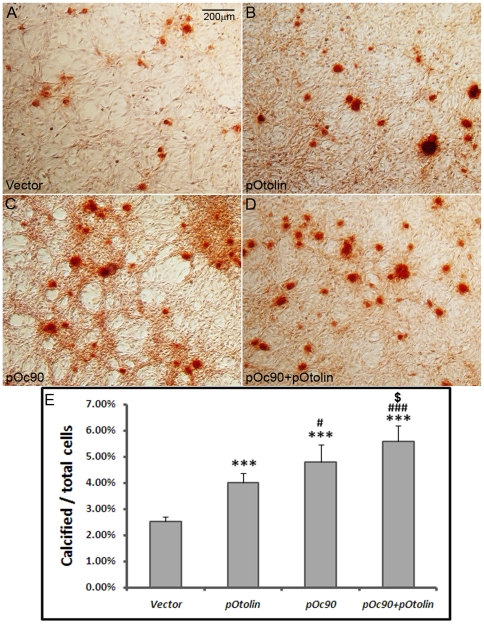
Oc90 and otolin augment ECM calcification. Inorganic calcium deposits were visualized with Alizarin Red S staining, and averaged percentages of cells with ECM calcification over total cells were compared for different constructs and vectors. Under the same inducing conditions, untransfected cells and cells transfected with empty vectors (**A**) had similar ratios of calcification nodules, but both had significantly lower ratios than those transfected with otolin (**B**), Oc90 (**C**), or Oc90+otolin (**D**) (Figure 5B, 5C or 5D vs. 5A, p<0.001 as denoted by ***, n = 3 experiments x 3 fields for each). **Figure 5E** shows histograms of the averaged percentages of cells with ECM calcification. Co-transfection of Oc90 and otolin had a synergistic effect on calcification. # and ### indicate p<0.05 and 0.001, respectively, when pOc90 or pOc90+pOtolin is compared with pOtolin. $ indicates p<0.05 when pOc90+pOtolin is compared with pOc90.

We then established a cell culture system to test whether the presence of Oc90 and/or otolin can augment matrix calcification. NIH/3T3 cells stably transfected with Oc90 and/or otolin showed intense ECM calcification after 5 days of induction with 0.5 mM Ca^2+^ and 2 mM Pi (Figure 5). The Ca^2+^ concentration used here is much lower than that used in osteoblast calcification studies (a minimum of 2 mM for the latter); the Pi concentration is slightly lower too (a minimum of 2.5 mM for the latter) [42]. In the figure, inorganic calcium deposits were visualized with ARS staining, and percentages of cells with ECM calcification over total cells were obtained in each sample. No calcification was seen in untransfected or transfected cells cultured in standard media without additional supplements of Ca^2+^ and Pi (Figure S2). Under inducing conditions, ratios of cells with ECM calcification were similar between untransfected cells and cells transfected with empty vectors. Among cells stably transfected with otolin, an average of 4.0±0.4% had calcification nodules, as compared to 2.5±0.2% for those transfected with the empty vector under inducing conditions (Figure 5A & 5B) (p<0.001, n = 3 experiments x 3 survey fields in each). The effect of Oc90 on calcification (4.8±0.7%) was significantly stronger than that of otolin (p<0.05). Co-transfection of Oc90 and otolin had a synergistic effect on calcification, with 5.6±0.6% cells showing matrix calcification (p<0.05 vs. Oc90 transfectants, and p<0.001 vs. otolin transfectants). Due to the large numbers of total confluent cells in each view field, the percentages of cells with calcification nodules became small, but the differences between Oc90 (or otolin or Oc90+otolin) transfectants versus empty vectors were visually obvious even without a microscope. Therefore, the presence of Oc90 and/or otolin significantly increases the propensity of the extracellular matrix to calcify.

### Structural analysis and molecular modeling predict critical features that may underlie the Oc90-otolin interaction and Ca^2+^-sequestering ability

**Figure 6:**
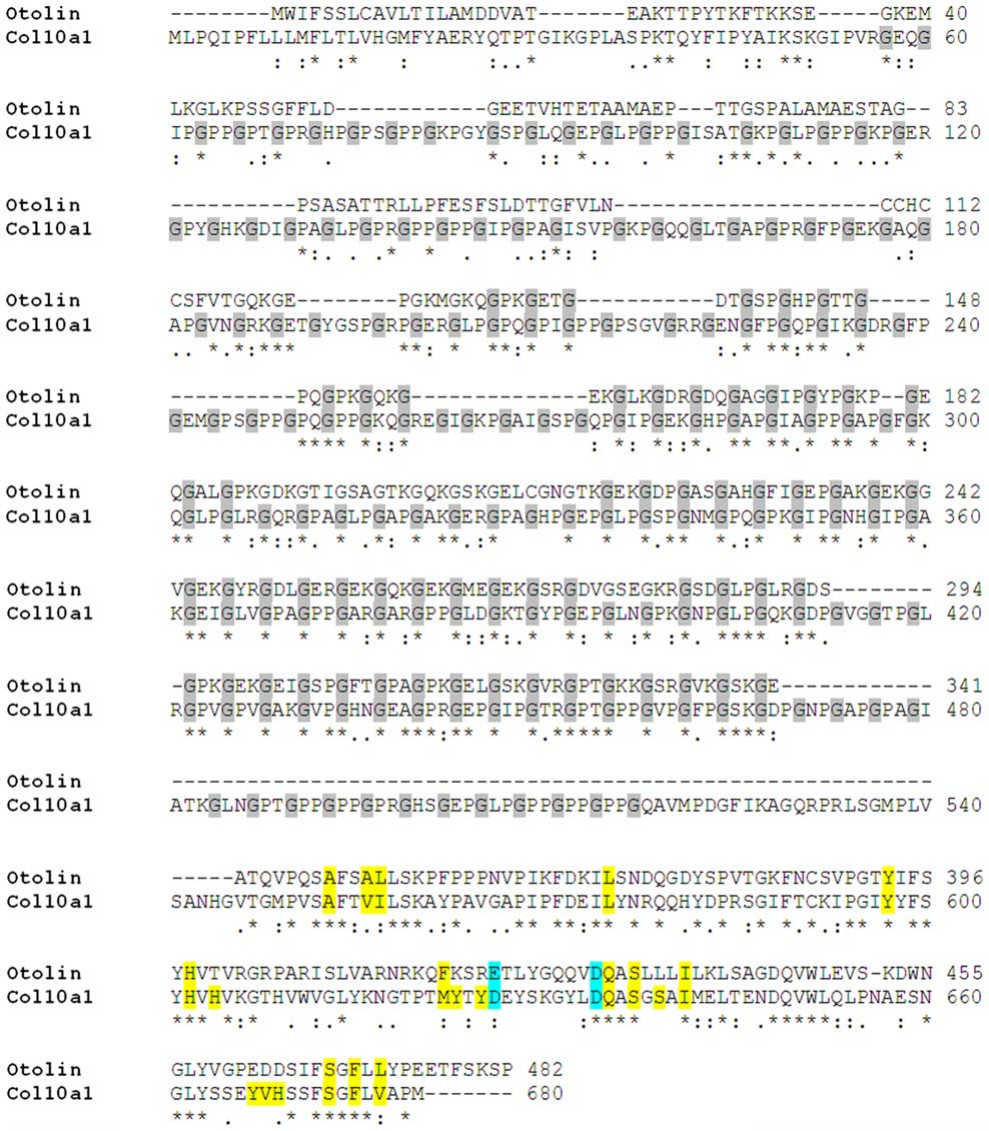
Alignment of murine otolin and collagen X-a1 (Col10a1). The collagen-like domain, aka triple helix (TH) domain, of otolin and Col10a1 have a repeating (Gly-X-Y)_n_ (the shaded ‘G’s denote Glycine), where X is frequently Proline and Y hydroxyproline. The TH domain starts at residue 130 and ends at residue 342 in otolin. The C-terminal globular C1q (gC1q) domain starts at residue 343 in otolin. In collagen, this domain is also known as the non-collagenous NC1 domain. The two D's (blue) denote aspartic acids in Col10a1 that provide the ligands for a cluster of four Ca^2+^ ions. These residues are conserved in otolin. The inter-subunit contacts are almost entirely hydrophobic with key contact residues highlighted in yellow. Most of these residues are conserved in otolin. Residues 1–23 in otolin and 1–18 in Col10a1 are the signal peptides, respectively; residues 19–56 in Col10a1 is the non-collagenous NC2 domain, and 24–36 in otolin is the residual NC2-like domain. * : . indicate identical residues, conservative and semi-conservative variations, respectively, between otolin and Col10a1.

Our recent structural analysis of Oc90 shows that some functionally critical features can be modeled and predicted from known crystal structures of their homologs and paralogs [9], although a co-crystal structure is necessary to know if the Oc90-otolin complex goes through a significant conformational change upon contact. Here we utilized the high degree of sequence similarity between otolin (Swiss-Prot id Q4ZJM7) and type X collagen (Col10a1) (Swiss-Prot id Q05306) (Figure 6) and modeled the tertiary structures. Both proteins belong to a subgroup of the collagen family and the C1q super-family (the two families have overlapping subgroups). Otolin has 74 characteristic Gly-X-Y repeats and Col10a1 has 129 Gly-X-Y repeats in the collagen-like domain, where X is frequently Proline (Pro) and Y hydroxyproline in collagen. Otolin has fewer Pro residues, with 36 total Pro and 15 in Gly-X-Y repeats. Nevertheless, molecular modeling shows that the TH domain of otolin resembles the structure of human COL10A1 [43]: it also has a linear architecture with a flexible and dynamic structure and will likely form a triple helix.

**Figure 7:**
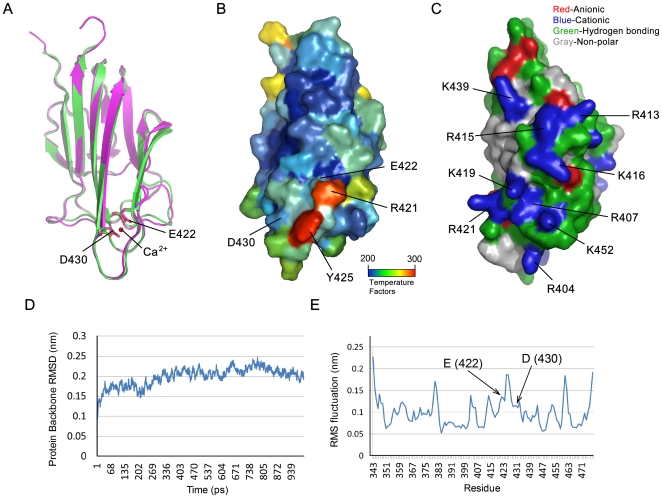
Molecular modeling of the C1q domain of otolin. (**A**) The tertiary structures of Col10a1-C1q domain (green) and otolin-C1q domain (magenta) are nearly super-imposable. The modeled structure is consistent with the crystal structure of human COL10A1. Ca^2+^-binding residues are labeled and projected as sticks. (**B**) The surface of the otolin-C1q domain with temperature factors denoted in different colors. (**C**) Clustering of residues and the cationic surface of otolin-C1q domain. Residues are colored according to their electrical charges and hydrophobic nature. The nine key cationic residues are labeled and projected as sticks. (**D**) The protein backbone RMSD during 1ns simulation. After 400ps, the structure is stable. The 400–1000 ps range was used for computing the final structure. (**E**) The temperature factor (RMS fluctuation) of each residue. The residues with higher RMS fluctuation usually are exposed on molecule surface and may construct active sites. The Ca^2+^-binding residues in (A) are labeled.

Both otolin and Col10a1 have a highly conserved C-terminal globular C1q (gC1q) domain, sharing 68% amino acid sequence similarity to each other, with 55 identical (*), 20 conservative (:) and 18 semi-conservative (.) residues among a total of 136aa (Figure 6). The inter-subunit contacts in the trimer are almost entirely hydrophobic with key contact residues highlighted in yellow in Figure 6. Thirteen out of 20 residues in Col10a1 are conserved in otolin. Based on molecular modeling results, the tertiary structures of murine otolin and Col10a1 are nearly super-imposable (Figure 7A). Most importantly, the two critical aspartic acids (D, highlighted in Figures 6 & 7) that provide the ligands for a cluster of four Ca^2+^ ions in Col10a1 are conserved in otolin (E422 binds Ca^2+^ equally well). With other residues (R421 and Y425), they form a pocket with high temperature factors for Ca^2+^ binding (Figure 7B, E). Additionally, a stretch of nine positively charged residues is present nearby the pocket and surrounded by several hydrogen-bonding residues (Figure 7C), which constitutes a large cationic and hydrogen-bonding surface and should represent a putative functional site/region for Oc90 binding. Possible electrostatic interactions and hydrogen bonding between otolin and Oc90 is further supported by the clustering of anionic and hydrogen-bonding residues on the surface of a modeled Oc90 structure [44]. At the physiological pH, otolin has a net positive charge of 11.5 with a predicted pI of 9.2, and Oc90 has a negative charge of −23.0 with a predicted pI of 4.5. The non-polar residues may also facilitate the interaction of otolin with Oc90. Clustering of non-polar residues was also found on the surface of murine Oc90 [45] and bullfrog Oc22 [44], a homolog of Oc90 but with only one sPLA2-like domain.

Figure 7D shows the root mean square deviation (RMSD) of the backbone atoms along the trajectory, which is used to assess the structural stability of the homology models. The RMSD of the otolin-C1q domain (residues 343–482) shows that during simulation the structures quickly reached equilibrium and then they are fluctuated around an average structure. Good quality of the modeled structure is indicated by the low RMSD value (0.21 Å) of the time-averaged structure and a TM-score (estimated accuracy of the model) of 0.87. Figure 7E shows the RMS fluctuations used to map the surface residues of the modeled structure in Figures 7B and 7C. Low RMS fluctuations are indicative of tighter bound complexes, whereas high fluctuations suggest surface residues.

## Discussion

Otoconia-related balance disorders are prevalent. For example, benign paroxysmal position vertigo, a disease believed to be caused by otoconia dislocation with possible degeneration, is the most common form of dizziness. In order to uncover the molecular etiology of such disorders, we began by studying the mechanisms underlying otoconia development. In this study, we have demonstrated that Oc90 interacts with both domains of otolin to form the otoconial matrix framework, and this complex sequesters Ca^2+^ in the ECM for efficient calcification. These proteins have much higher levels of expression in the utricle and saccule than the cochlea or canal/ampulla, likely contributing to the spatial specific formation of bio-crystals. Based on what is known of bone matrix proteins, the affinity of the otoconial matrix proteins for Ca^2+^ is not expected to be high. However, the affinity of otoconial proteins does not need to be high for the proposed function to occur, as the micro-environmental Ca^2+^ level may be high due to the activities of nearby Ca^2+^ channels and pumps such as Atp2b2 (also called Pmca2, or plasma membrane Ca^2+^-transporting ATPase 2). ECM-immobilization of Ca^2+^ serves two important purposes: (1) The high ECM-Ca^2+^ content provides a reservoir for otoconia formation, maintenance and neogenesis, and (2) The ECM is able to reduce the micro-environmental Ca^2+^ that is extruded by ion pumps/channels, as high Ca^2+^ is not conducive for optimal hair cell function.

The interaction of Oc90 and otolin is not surprising, as its structural paralog sPLA2 can induce collagen deposition in the vascular wall [46]. Notably, several collagen-binding proteins (such as Sparc, integrin, osteopontin, bone sialoprotein, fibronectin, vitronectin, see Introduction for references) do not share any sequence similarity. Likewise, there is no sequence similarity between Oc90, Sc1 (or Sparc) and the predominant matrix protein in bony fish, otolith matrix protein (OMP), yet, all these otoconia/otolith proteins function similarly in calcification. For example, Sc1 is incorporated in the absence of Oc90 and partially compensate for the loss of Oc90 function [9]; OMP was found to recruit otolin [33], despite a lack of sequence similarity between Oc90 and OMP. This phenomenon points to the possibility that certain residues, motifs, and tertiary features may be necessary and sufficient for binding matrix proteins and calcite (or apatite) during otoconia/otolith formation. Indeed, Oc90, Sc1 (or Sparc) and OMP all share the following critical features that are faithfully conserved throughout evolution: (1) All are very acidic due to the large numbers of Glu and Asp residues. These residues confer a high affinity for calcium phosphate [47]; [48] and calcium carbonate. (2) All bind bivalent cations (e.g. Ca^2+^). (3) All bind otolin and/or other types of collagen [10]; [32]; [49]–[51]. The conserved functional domains of Oc90, Sc1 and Sparc have α-helices [9] that may be important for interacting with Ca^2+^, calcium carbonate/phosphate crystals or matrix proteins. (4) All are rich in Cys residues, which are predicted to form intra- and possibly inter-molecular disulfide bridges to form a rigid and stable framework for ordered mineral deposition. (5) All are glycosylated, another feature that endows the protein with an affinity for Ca^2+^. (6) Both Oc90 and OMP are expressed in all non-sensory epithelia of the developing otocyst [33]; [52].

The types of interactions (electrostatic, hydrogen-bonding, and hydrophobic) we predicted between Oc90 and otolin may also occur between the two proteins and KSPG. Corneal KSPG has been shown to have a somewhat rigid conformation and is stable, likely due to disulfide bonds [53]. Similar to other proteoglycans (i.e. HSPG) [54], KSPG interacts with collagen through electrostatic interactions. In addition, KSPG possesses significant surface hydrophobicity that allows it to bind lipid assemblages [53].

In the absence of Oc90, Sc1 or even some residual otoconial components (e.g. KSPG, osteopontin and fetuin-A) in the macular ECM may sequester Ca^2+^ for CaCO_3_ seeding albeit with reduced efficiency. Indeed, Sc1 can enhance matrix calcification as well [9]. Possible functional redundancy among a few otoconins *in vivo* is further suggested by the fact that several other otoconins, in addition to Oc90, can also regulate crystal growth and morphology *in vitro* [44]; [45] but are dispensable for otoconia formation *in vivo* (i.e. osteopontin, fetuin-A). Apparently, this redundancy cannot efficiently compensate for the loss of Oc90 *in vivo* [9]; [10].

Alternatively, the insignificance of the other proteins in otoconia formation may be due to their low abundance. However, Oc90 has relatively low abundance in the zebrafish otolith (known as Otoc1) [55], and yet Oc90 knockdown mutants show a range of phenotypes including missing, aberrant, small to normal otoliths [55]. These phenotypes are more severe than those of OMP1 morphants [33]; [55], suggesting that zOc90 (Otoc1) is essential for the early stages of otolith development (i.e. crystal seeding) whereas OMP regulates crystal growth. The onset of Oc90 expression is the earliest among all otolith/otoconia proteins in zebrafish and mice (before E9.5 in mice) [7]; [8]; [55], much earlier than the onset of any activities of ion channels/pumps. Oc90 then recruits other components at the time of their expression to form the organic matrix prior to onset of calcification [10]. Thus, the structure and function of Oc90 is conserved from bony fish to mice (two model systems whose otoconia/otolith are the most studied) regardless of the abundance of the protein in each species.

Our data show that otolin shares high structural and functional similarity to Col10a1. Conservation of the Col10a1 features in otolin may render the protein extremely stable. Native Col10a1 forms a strong, non-covalent trimer that is not disrupted even under reducing agents [43]. The trimers then form a higher order aggregate [43]. The crystal structure of human COL10A1 shows an absence of disulfide bridges [56]–[58]. Like Col10a1, otolin only has 1 Cys residue, but it may be unnecessary to form inter-molecular disulfide bridges as well.

Although both NC1 and NC2 domains of Col10a1 were proposed to be important for intermolecular interactions and the formation of extracellular supramolecular assemblies [59], studies have addressed mostly the structure and function of the C-terminal NC1 domain. The NC2 domain in otolin is even shorter (13 residues as compared to 37 in Col10a1, excluding signal peptide) and less conserved than the rest of the protein, therefore, we focused on the NC1 domain in our analysis.

**In summary**, the present study has uncovered an important mechanism responsible for the specific recruitment of otoconia matrix components for optimal and spatial specific otoconia formation. These components, especially the predominant otoconin Oc90, are critical to sequester Ca^2+^ for normal otoconia formation. Structural analysis suggests important features underlying such functions. In the process, we have developed a sensitive assay utilizing ratiometric fura-2 to quantify the matrix-Ca^2+^ content as an indicator of its potential to calcify. Future studies will identify additional mechanisms (e.g. the role of regulatory proteins such as Otop1, Nox3 and Noxo1) that likely exist to determine spatial specific biomineralization. It should be noted that, after much endeavor of the bone field, mechanisms underlying spatial restriction of mineralization to bone are still controversial and are far from being fully resolved. It is thus anticipated that a large amount of continuous effort will be required to achieve similar goals in the otoconia field as well.

## Supporting Information

Figure S1 **Co-immunoprecipitation of Oc90 and otolin with KSPG.** (**Top**) KSPG antibody (labeled as α-KSPG) pulled down Oc90 from extracts of otoconia (O) and vestibular epithelia (Ve) but not when mouse IgG was present. (**Bottom**) KSPG antibody pulled down otolin from transfected HEK293 cells (labeled as “cell”) and vestibular epithelia, but not when mouse IgG was present.
(TIF)

Figure S2 **No calcification without Ca^2+^ and Pi.** No calcification was seen in any of the cells without supplemental Ca^2+^ and Pi (pOtolin transfectants are shown here). ARS staining was performed in the same way as for Figure 5.
(TIF)

Method S1 **Co-immunoprecipitation (co-IP) of Oc90, otolin and KSPG for Figure S1.**
(DOCX)
