# Supplementary figures and images for: Matrix Recruitment and Calcium Sequestration for Spatial Specific Otoconia Development
Source: PLoS One. 2011 May 31;6(5):e20498. doi: 10.1371/journal.pone.0020498 (PMC3105080; doi:10.1371/journal.pone.0020498)

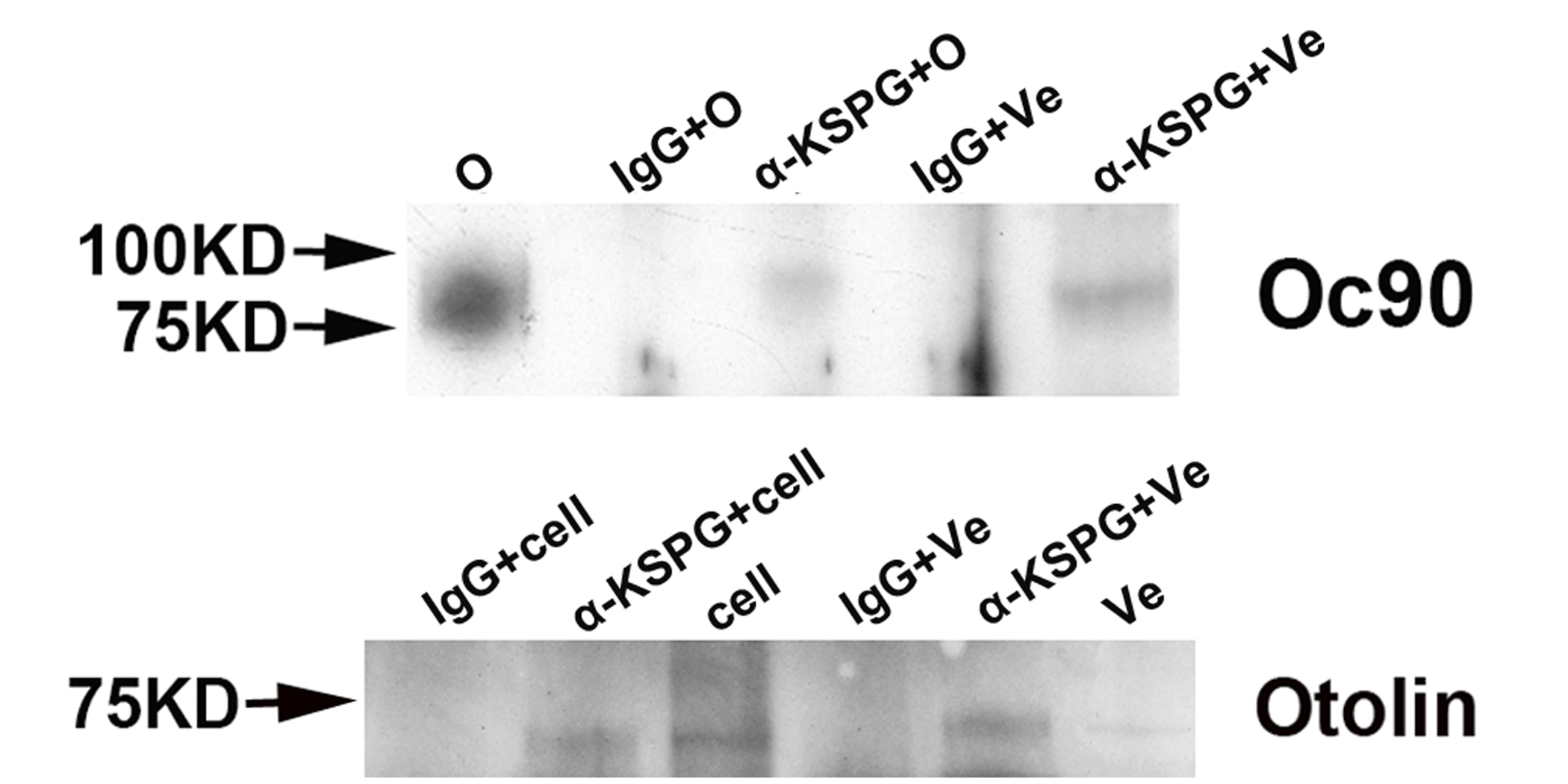

Supplement: Figure S1 — Co-immunoprecipitation of Oc90 and otolin with KSPG. (Top) KSPG antibody (labeled as α-KSPG) pulled down Oc90 from extracts of otoconia (O) and vestibular epithelia (Ve) but not when mouse IgG was present. (Bottom) KSPG antibody pulled down otolin from transfected HEK293 cells (labeled as “cell”) and vestibular epithelia, but not when mouse IgG was present. (TIF) [file pone.0020498.s001.tif]

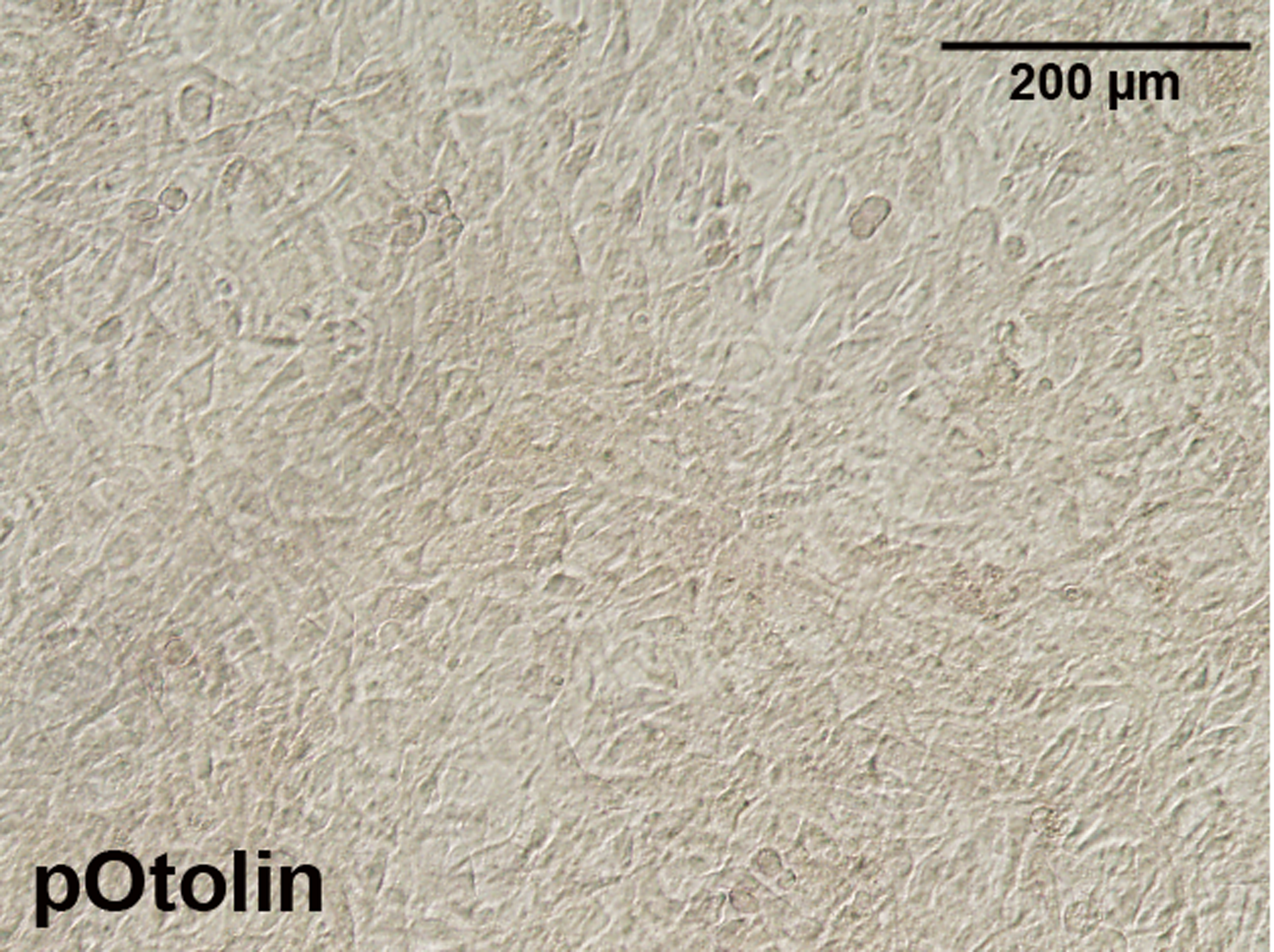

Supplement: Figure S2 — No calcification without Ca2+ and Pi. No calcification was seen in any of the cells without supplemental Ca2+ and Pi (pOtolin transfectants are shown here). ARS staining was performed in the same way as for Figure 5. (TIF) [file pone.0020498.s002.tif]
