# Supplementary material for: Matrix Recruitment and Calcium Sequestration for Spatial Specific Otoconia Development
Source: PLoS One. 2011 May 31;6(5):e20498. doi: 10.1371/journal.pone.0020498 (PMC3105080; doi:10.1371/journal.pone.0020498)
Supplement: Method S1 — Co-immunoprecipitation (co-IP) of Oc90, otolin and KSPG for Figure S1. (DOCX) [file pone.0020498.s003.docx]

**Method S1. Co-immunoprecipitation (co-IP) of Oc90, otolin and KSPG for Figure S1**

Cell lysates were prepared from stable otolin transfectants of NIH/3T3 cells. After several washes in PBS, the cultured cells were re-suspended in 1 ml IP buffer (1% Brij 97, 10mM Tris:HCl pH 7.4, 150 mM NaCl, 0.5 mM CaCl_2_, 0.5 mM MgCl_2_ and protease inhibitors), and gently rocked on ice for 10 min. Samples were then centrifuged at 13,000 rpm (16,000 g) for 10 min at 4°C, and supernatants were used in co-IP reactions below.

Tissue lysates were prepared from dissected utricles/saccules (pooled; labeled as “Ve” for vestibule in Figure S1) and otoconia after lysis with 240 µl IP buffer and 120 µl 0.5M EDTA (pH8.0) with gentle rocking for 4h at 4ºC. Tissue lysates were centrifuged at 13,000 rpm (16,000 g) for 10 min at 4ºC and supernatants were transferred to a Centrion column (Millipore) to remove EDTA.

To coat protein A-sepharose beads (Sigma) with anti-KSPG (or mouse IgG as control), the beads were swelled and washed in PBS at 4ºC. Five micrograms of anti-KSPG (or mouse IgG) and 200 µl of cold PBS were added to 30 µl beads, and incubated overnight at 4ºC with gentle rocking. After washes with cold PBS and then with cold IP buffer, 200 µl cell lysates (or 50 µl tissue lysates) and 150 µl IP buffer were added to the coated beads. Samples were incubated overnight at 4ºC with gentle rocking, followed by several washes with IP buffer, then with IP buffer plus 58.44mg/ml NaCl, and with IP buffer again. Samples were re-suspended in 60 µl of 1X loading buffer containing 2% SDS, boiled for 5 min and centrifuged at 13,000 rpm (16,000 g) for 10 min at 4ºC. Aliquots (20 µl) of supernatants were analyzed by Western blotting as described in the manuscript.
